# A community survey of the pattern and determinants of household sources of energy for cooking in rural and urban south western, Nigeria

**Published:** 2012-05-03

**Authors:** Olufemi Olumuyiwa Desalu, Ololade Olusola Ojo, Ebenezer Kayode Ariyibi, Tolutope Fasanmi Kolawole, Ayodele Idowu Ogunleye

**Affiliations:** 1 University of Ilorin Teaching Hospital Ilorin, Nigeria; 2 Federal Medical Centre, Ido-Ekiti, Nigeria

**Keywords:** Household energy, biomass fuel, indoor pollution, Lung, Nigeria

## Abstract

**Introduction:**

The use of solid fuels for cooking is associated with indoor pollution and lung diseases. The objective of the study was to determine the pattern and determinants of household sources of energy for cooking in rural and urban South Western, Nigeria.

**Methods:**

We conducted a cross sectional study of households in urban (Ado-Ekiti) and rural (Ido-Ekiti) local council areas from April to July 2010. Female respondents in the households were interviewed by trained interviewers using a semi-structured questionnaire.

**Results:**

A total of 670 households participated in the study. Majority of rural dwellers used single source of energy for cooking (55.6%) and urban dwellers used multiple source of energy (57.8%). Solid fuel use (SFU) was higher in rural (29.6%) than in urban areas (21.7%). Kerosene was the most common primary source of energy for cooking in both urban and rural areas (59.0% vs.66.6%) followed by gas (17.8%) and charcoal (6.6%) in the urban areas, and firewood (21.6%) and charcoal (7.1%) in the rural areas. The use of solid fuel was strongly associated with lack of ownership of dwellings and larger household size in urban areas, and lower level of education and lower level of wealth in the rural areas. Kerosene was associated with higher level of husband education and modern housing in urban areas and younger age and indoor cooking in rural areas. Gas was associated with high income and modern housing in the urban areas and high level of wealth in rural areas. Electricity was associated with high level of education, availability of electricity and old age in urban and rural areas respectively.

**Conclusion:**

The use of solid fuel is high in rural areas, there is a need to reduce poverty and improve the use of cleaner source of cooking energy particularly in rural areas and improve lung health.

## Introduction

Cooking in a household involve the use of solid fuel and nonsolid fuel [1]. The solid fuel consists of coal which is a fossil fuel and biomass fuel (BMF) like wood, charcoal, dung and crop residues. Worldwide, more than three billion people depend on solid fuels, including biomass (wood, dung and agricultural residues) and coal, to meet their most basic energy needs: cooking, boiling water and heating [1]. The non solid fuel consists of kerosene, liquefied petroleum gas (LPG), gas and electricity [1, 2]. The use of some solid fuels has been associated indoor pollution and unsafe levels of toxic emission [3].

BMF usage is associated with high levels of indoor air pollution and an increase morbidity and all-cause mortality both in adults and children [4–7]. Indoor air pollution accounted for 1.5 to 2 million deaths per year worldwide, half of them occurring in children younger than five years due to acute respiratory infections (ARI), but also in women due to chronic obstructive pulmonary disease (COPD) and lung cancer [8–10]. Close to 50% of the world population, around 3 billion people, uses biomass fuels as their primary source of domestic energy for cooking [1]. In Nigeria, the percentage of households using solid fuel is high (70%), including 86 percent of households in rural areas and 42 percent of households in urban areas. Among the households that reported use of solid fuel for cooking, the majority (94 percent) were using an open fire/stove without a chimney or hood [11]. Studies in South West Nigeria and Ghana have found that there is an increase in the risk of respiratory morbidity and chronic bronchitis in those using biomass fuels [12, 13]. The cost implication of managing a chronic respiratory condition resulting from exposure to indoor pollution in a resource poor setting is huge therefore, it's important to focus on the preventive measures. For an effective prevention and intervention against indoor pollution from household fuel, there is a need to identify the pattern of household cooking fuel and the factors that influence the choice of household cooking fuel. These determinants of household cooking fuel have been mostly established in some urban settings in some countries [13, 14]. Therefore to have an insight about the pattern consumption in the both rural and urban setting, this study was conducted to establish the pattern and determinants of household sources of energy for cooking in rural and urban south western Nigeria and also to discuss the implication for lung health and possible interventions.

## Methods

### Study design and setting

This was a descriptive cross sectional study of households in Ekiti State, South Western Nigeria that was carried out over three months from April 2010 to August 2010. The country is the most populous black nation in the world with a population of 145 million people and is composed of more than 250 ethnic groups; the male: female ratio is 1.04:1, fifty three percent of population lived in the rural area while 47% lived in the urban areas [15, 16]. This study was carried out in Ado-Ekiti, the capital of Ekiti State, which is the urban setting and Ido-Osi local government area in Ekiti state Nigeria which is the rural setting. We selected one urban and one rural local government area to have a true representation of the study population. The study area met the United Nation (UN) definition of urban settlements for Nigeria: which is defined as towns with 20,000 inhabitants or more whose occupations are not mainly agrarian [16, 17]. Majority of the inhabitants in this rural area are farmers while that of Ado-Ekiti are civil servant and traders.

### Study sample

The households were selected by multistage stratified sampling method in the study areas. The local government areas consist of electoral wards that were defined as clusters and a sample frame containing the list of clusters was constructed by the investigators. Of these, 3 clusters were selected by simple random sampling in Ado-Ekiti and Ido-Osi respectively. Subsequently the households in each cluster were randomly selected. The women in each household were informed about the study by our trained interviewers and one woman per household was selected to participate in the study. In polygamous family the most senior wife was selected due to their wealth of information on the family. The inclusion criteria were women (1) aged 18 years and above (2) who gave informed verbal or written consent (3) resided in Ekiti state for one year. Men were not interviewed because in the Nigerian society, culturally it is not a man's responsibility to cook.

### Survey instrument

The survey instrument was a semi-structured questionnaire that was prepared from questions taken from similar studies [13, 14, 18].This questionnaire was semi-structured in to order accommodate qualitative and quantitative information's. It was administered by three female interviewers who had one day training on method of questionnaire administration prior to the field survey. The questionnaire consist of three sections, the first section contain questions on demographic information, household size, family monthly income, ownership of household items and dwellings, type of home, and availability of home utilities (water and electricity). The second part contain questions on the primary or dominant fuel used for cooking by the household, cost of cooking fuel per week, reason(s) for choosing cooking fuel, frequency of cooking their regular local food and average time spent cooking per day which was multiplied by 7. The third part had questions on stove and kitchen type and ventilation, awareness of the harmful effect of inhaling smoke from solid fuel on the lungs.

### Ventilation coefficient

The population exposed to solid fuel was calculated from the coefficient of ventilation which was based on two ventilation-related factors: (i) improved stoves; and (ii) outdoor cooking [17]. Improved stoves were characterized by the presence of a flue, chimney, or hood, which can markedly reduce exposures. A ventilation coefficient of 1.00 was applied to the population that uses traditional stoves and ventilation coefficient of 0.25 to the population that uses improved stoves, or cooks outdoors. Population exposed to SFU = (population size) × (% of households using solid fuels with traditional stoves) × (ventilation coefficient of 1.00) + (population size) × (% of households using solid fuels and either improved stoves or cooking outdoors) × (ventilation coefficient of 0.25).

### Wealth index

We collected household information on the type of housing material used for the floors, source of electricity and drinking water supply, toilet facility, source of cooking fuel and owning a means of transportation (car, bicycle, motor cycle, trucks and camels). In addition, we also collected information on household assets like farm land, domestic animals and numbers of rooms and people sleeping in each room in the household as well as having a domestic worker not related to the head of the household. We scored those items using the items score previously used to create a wealth index of the households interviewed in the Health Nutrition and Population Survey of Nigeria 2003[11]. The total of the item scores was used to calculate the total household assets score and asset Index value. A Wealth Quintile was produced from the asset Index value and was divided into five wealth quintile according to the asset Index value: households were categorized as low quintile if asset Index value was ( Low to −0.87509), second quintile ( −0.87509 to −0.63349),third quintile ( −0.63349 to −0.09805), fourth quintile (−0.09805 to 0.92047), High quintile (=0.92047) [11].

### Operational definitions

Primary cooking fuel was defined as the fuel used mainly by a household for cooking [17]. Secondary cooking fuel was defined as the fuel used for supplemental purposes, as a backup for fuel-specific cooking activities by a household. Open fire or stove was defined as a simple arrangement of three rocks, a U-shaped hole in a block of clay, or a pit in the ground, or in poorly functioning earth or metal stoves [8]. Closed stove was defined as a metal stove which is air tight with a chimney. Traditional stove was defined as a simple arrangement of three rocks, a U-shaped hole in a block of clay, or a pit in the ground, or in poorly functioning earth or metal stoves without flue, chimney, or hood [8, 17].

Improved stove was defined as a simple arrangement of three rocks, a U-shaped hole in a block of clay, or a pit in the ground, or in poorly functioning earth or metal stoves with flue, chimney, or hood. [8, 17].

### Data analysis

The data were analyzed using Statistical Package for Social Science (SPSS version 15 Chicago IL Inc). The general characteristics of the study population were determined by descriptive statistics. The median and interquartile range were adopted for some variables because of the skewedness of their distribution.

The samples were stratified into two categories on the basis number of fuel used for cooking (1) single and multiple fuel - primary and secondary (2) dwellings of respondents into rural or urban. The chi square was used determine the significance of association between categorical and continuous variables according to rural and urban dwellings. In order to establish the factors that influence the choice of cooking fuel, the six categories of fuel were merged into four consisting of solid fuel, kerosene gas and electricity. The five quintiles were merged to three because some of the variables were not enough for analysis. These were low wealth index (poor family), middle (average family) and high wealth index (rich family).

Spearman coefficient of correlation (r) was used to determine the factors that influence the choice of household cooking fuel. P values

### Ethical approval

The study was approved by the ethics and research review committee of the Federal Medical Centre of Ido-Ekiti, Nigeria.

## Results

### General characteristics of the study population

At the end of the study, 759 households were approached but only 670 fully completed the study giving a response rate of 88.3% in both rural and urban areas. Out of the 670 households, 338 (50.4%) were recruited from the rural areas and 332(49.6%) from the urban areas. The mean age of the respondents was 43.1 ± 15.1 years and the median household income was higher in the urban areas (N 30,000 vs. N17, 000). The median household size was higher in the rural areas (4.0 vs. 2.0 per household) than the urban areas. Majority of the subjects who had low wealth index were in the rural areas and those who in the middle to high wealth index were in the urban areas (Table 1).

**Table 1:** General characteristics of the study population

Characteristics	Urban = 332	Rural = 338	All = 670
**Mean Age (SD)**	40(11)	46(18)	43(15)
Household income (Naira)*	30,000(1000-350,000)	17000((1500-80,000)	25,000(1,000-350,000)
Population per household*	2(1-11)	4(1-13)	3(1-13)
**Educational attainment**			
None /Elementary	68(20.5)	167(49.4)	235(35.1)
Secondary	108(32.5)	121(39.8)	229(34.2)
Tertiary	156(47.0)	50(14.8)	206(30.7)
**Spouse Educational attainment**			
None /Elementary	56 (16.9)	52(15.4)	108(16.1)
Secondary	112(33.7)	140(41.4)	252(37.6)
Tertiary	164(49.4)	146(43.2)	310(46.3)
**Marital status**			
Married	234(77.0)	232(71.2)	476(74.0)
Single/divorce/widow	90(33.0)	104(28.8)	194(26.0)
**Religion**			
Christianity	235(70.8)	271(80.2)	506(75.5)
Others	97(29.2)	67(19.8)	164(24.5)
**Wealth index**			
Low	185(55.7)	299(88.5)	484(72.2)
Middle	102(30.7)	39(11.5)	141(21.0)
High	45(13.6)	0(0.0)	45(6.7)
Household utility-Electricity*			
Yes	296(89.2)	309(91.4)	605(90.3)
No	36(10.8)	29(8.6)	65(9.7)
**Household utility-Water**			
Yes	238(71.7)	200(52.9)	438(65.4)
No	94(28.3)	138(40.8)	232(34.6)
**Housing type**			
Modern houses	110(33.1)	37(10.9)	147(21.9)
Traditional houses	222(66.9)	301(89.1)	521(78.1)
**Awareness of adverse effect of solid fuel on the lung**			
Yes	216(69.2)	328(98.8)	544(81.2)
No	105(30.8)	21(1.2)	126(18.8)

* Data are median (Interquartile range); Other data are in frequency (%)

### Pattern of household sources of energy for cooking, kitchen characteristics and stove types in urban and rural areas

Three hundred and forty three households (51.2%) used two or more sources of energy for cooking while 327(48.2%) used a single source energy for cooking. The use of multiple source of energy for cooking was more common in the urban areas than the rural areas (57.8% vs. 44.4%) while the use of a single source of energy was more common in the rural areas (42.2% vs.55.6%). Kerosene(59.0%), gas(17.8%) and coal (6.6%) were the leading primary sources of cooking energy in the urban areas and while kerosene(66.6%), wood(21.6%) and coal(7.1%) were the leading primary sources of cooking energy in rural areas. The most common secondary source of cooking energy in urban areas was gas (25.0%) and in the rural areas it was wood (16.9%) (Table 2). The prevalence of solid fuel use (SFU) in this study was 26.9%; it was higher in the rural than in the urban areas (21.7% vs.32.0%). After adjusting for the ventilation coefficient which was based on type of cooking stoves and kitchen location, 172(25.2%) were actually exposed to solid fuel and (29.6%) of them were rural dwellers and 21.7% were urban dwellers.

**Table 2:** Pattern of household sources of energy for cooking, kitchen characteristics and stove types among urban and rural dwellers

Characteristics	Urban = 332	Rural = 338	All =670
**Cooking fuel sources**			
Single	140(42.2)	188(55.6)	328(49.0)
Multiple	192(57.8)	150(44.4)	342(51.0)
**Primary cooking fuel**			
Agric waste	20(6.0)	11(3.2)	31(4.6)
Wood	30(9.0)	73(21.6)	103(15.4)
Coal	22(6.6)	24(7.1)	46(6.9)
Kerosene	196(59.0)	225(66.6)	421(62.8)
Gas	59(17.8)	2(0.6)	61(9.1)
Electricity	5(1.5)	3(0.9)	8(1.2)
**Secondary cooking fuel**			
None	140(42.2)	188(55.6)	328(49.0)
Agric waste	7(2.1)	4(1.2)	11(1.6)
Wood	34(10.2)	57(16.9)	91(13.6)
Coal	25(7.5)	35(10.4)	60(9.0)
Kerosene	43(13.0)	5(1.5)	48(7.2)
Gas	83(25.0)	48(14.2)	133(19.6)
Electricity	0(0.0)	1(0.3)	1(0.1)
Stove type			
Traditional(open fire/stove)	72(21.7)	103(30.5)	175(26.1)
Improved traditional	0(0.0)	5(1.5)	5(0.8)
Kerosene stove	196(59.0)	225(66.6)	421(62.8)
Gas stove	59(17.8)	2(0.6)	61(9.1)
Electric stove	5(1.5)	3(0.9)	8(1.2)
**Kitchen location**			
Indoor	93(28.0)	81(24.0)	174(26.0)
Outdoor	156(47.0)	178(52.8)	334(49.9)
Separate building	83(25.0)	78(23.2)	161(24.1)
**Kitchen ventilation**			
Good	220(66.3)	74(21.9)	294(42.6)
Poor	112(33.7)	264(78.1)	376(57.4)
Household Cooking time per week(hrs)*	14(7-56)	28(7-70)	28(7-70)
**Exposure to solid fuel**	72(21.7)	100(29.6)	172(25..2)

* Data are median (Interquartile range). Other data are in frequency (%)

### Distribution of household sources of cooking energy according to the household wealth

**Figure 1:**
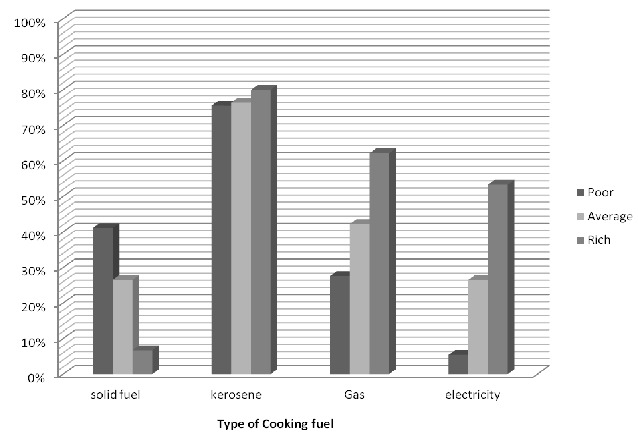
Choice of primary cooking energy in the urban area according to household wealth

**Figure 2:**
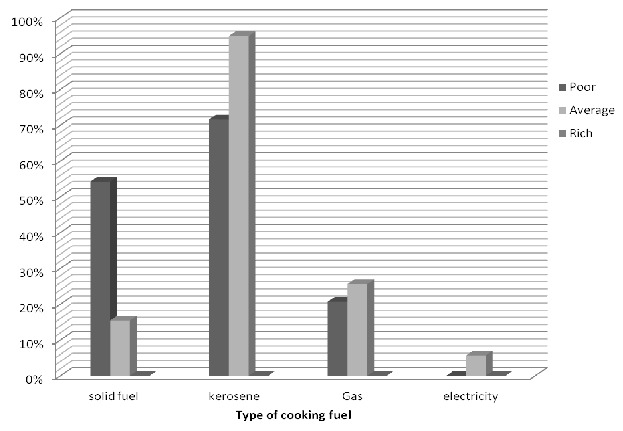
Choice of primary cooking energy in the rural area according to household wealth

In the rural areas, the use of solid fuel like wood, agric waste, dung and charcoal was higher in the poor families (Figure 1). In the urban areas, the use of solid fuel was also higher in the poor families and the use of gas and electricity were higher in rich families (Figure 2).

### Factors influencing the choice of cooking energy in urban and rural areas

Spearman coefficient of correlation (r) was used to determine the factors that influence the choice of household cooking fuel. Definition of the variables of the correlation coefficient is shown below (Table 3).

**Table 3:** Definition of the variables used in Spearman correlation coefficient test

Variables	Unit of account:	Modalities of variables	
Age of respondent	Years in number	(quantitative)	
Education of respondent	None/Primary -1	Secondary-2	Tertiary -3
Husband education	None/Primary -1	Secondary-2	Tertiary -3
Marital status	Married-1	Separated/divorce-2	Single -3
Household income	Income in Nigerian	naira (NGN)	(quantitative)
Household Wealth index	Lower-1	Middle -2	High -3
Household size		(quantitative)	
Ownership of dwelling	Owner -1	Otherwise-0	
Housing type	Modern -1	Otherwise -0	
**Household utility**			
Electricity	Yes -1	Otherwise -0	
Water	Yes -1	Otherwise -0	
Cooking frequency		(quantitative)	
Cooking time	Hours	(quantitative)	
Kitchen location	outdoor /separate from the sleeping room /Parlour-1	Indoor, Living areas/sleeping room or corridors-0	
Awareness of health effect of solid fuel on the lung	Yes -1	Otherwise -0	
Availability of fuel	Yes -1	Otherwise -0	
Affordability of fuel	Yes -1	Otherwise -0	
Seasons of the year	Yes -1	Otherwise -0	

The choice of solid fuel as source of cooking energy in urban areas were significantly associated with low level of education, lack of ownership of dwelling, large household size, residing in traditional houses, availability of water and awareness of adverse effect of wood smoke on the lung (Table 4). In the rural household the solid fuel use was associated lower level of wealth, lower level of education, residing in traditional houses and availability of solid fuel (Table 5).

**Table 4:** Determinants of household sources of energy for cooking in urban areas

Determinants	Solid fuel (r)	Kerosene (r)	Gas (r)	Electricity (r)
Age	+0.01	-0.05	+0.05	+0.01
Education of respondent	-0.29*	+0.07	+0.14	+0.34*
Husband education	-0.14	+0.26*	+0.15	+0.10
Marital status	+0.05	-0.17*	-0.03	+0.08
Household income	-0.05	-0.07	+0.41*	+0.13
Household Wealth index	-0.25	-0.05	+0.19*	-0.15
Household size	+0.31*	-0.03	+0.17*	-0.06
Ownership of dwelling	+0.34*	-0.23*	+0.13	+0.08
Housing type	-0.26*	+0.25*	+0.28*	+0.80
**Household utility**				
Electricity	+0.08	-0.06	-0.04	-0.16*
Water	+0.13*	-0.10	-0.07	-0.25*
Cooking frequency	-0.10	+0.20*	-0.78	-0.14
Cooking time	-0.07	+0.08	-0,03	-0.06
Kitchen location	+0.01	+0.02	-0.09	-0.07
Awareness of health effect of solid fuel on the lung	+0.12*	-0.03	-0.12	-0.24*
Availability of fuel	+0.11	+0.04	+0.11	+0.20*
Affordability of fuel	+0.02	+0.10	+0.18*	+0.07
Season of the year	+0.01	+0.04	+0.11	+0.07

* P < 0.05, r-Spearman correlation coefficient

**Table 5:** Determinants of household sources of energy for cooking in rural areas

Determinants	Solid fuel (r)	Kerosene (r)	Gas (r)	Electricity (r)
Age	+0.10	-0.14*	-0.05	+0.23*
Education of respondent	-0.28*	+0.13	+0.08	+0.14
Husband education	-0.08	+0.05	+0.14	+0.04
Marital status	+0.03	-0.01	-0.05	-0.06
Household income	-0.19	+0.16	-0.13	-0.07
Household Wealth index	-0.28*	-0.07	+0.33*	+0.04
Household size	+0.03	+0.04	+0.01	+0.02
Ownership of dwelling	+0.05	+0.02	-0.07	-0.07
Housing type	-0.17*	+0.06	-0.03	+0.09
**Household utility**				
Electricity	+0.08	+0.01	-0.02	-0.06
Water	+0.04	+0.62	-0.06	+0.11
Cooking frequency	+0.11	+0.05	+0.07	-0.81
Cooking time	+0.05	+0.05	+0.07	+0.02
Kitchen location	+0.19	-0.17*	-0.13*	+0.24*
Awareness of health effect of solid fuel on the lung	+0.06	+0.02	-0.01	+0.01
Availability of fuel	+0.17*	+0.07	+0.01	+0.20
Affordability of fuel	+0.05	+0.09	+0.11	+0.09
Seasonal of the year	+0.12	+0.02	+0.05	+0.01

* p < 0.05;, r-Spearman correlation coefficient

Kerosene use was positively associated with the higher level of education, residing in modern houses, marriage and increased frequency of cooking staple food and lack of ownership of dwellings in the urban households while in the rural household it was associated with kitchen location.

The use of gas was associated with household income, residing in modern houses, higher level of household wealth and larger size of the household in the urban areas and high level of household wealth and kitchen location in the rural areas. Electricity use was associated with increased age in the rural areas, and higher level of education, availability of home utilities like water and electricity in urban areas.

## Discussion

This was a comparative study of the household consumption pattern and determinants of cooking energy among rural and urban dwellers in South Western Nigeria. The majority of the households in urban areas used multiple sources of energy for cooking which was different to the rural areas where most household used single source of cooking energy. This observation is in contrast to a study in rural India where most of the household reported the use of multiple sources of energy [17]. The adoption of multiple cooking energy may be attributed to numerous exigencies that warranted the households to devise various alternatives to meet up their energy requirement, and possibly reduce their expenditures on cooking energy.

The most common primary cooking energy in both urban and rural areas was kerosene; firewood and charcoal were the second and third leading sources of cooking energy in the rural areas while in the urban areas it was gas and electricity. This pattern of cooking energy corroborates the result of other studies conducted in Nigeria [19–22]. In urban areas of five West African countries, charcoal is the predominant fuel [13, 14]. Generally, the global trend in the choice of household cooking energy is a function of geographical variation in the level of urbanization, living standard, and climate, socioeconomic and cultural factors. Other factors include shortages of particular fuels, lack of a distribution network, and failures of the distribution system [23, 24].

This study also found out that the population of people actually using and exposed to solid fuel was higher in the rural areas than in the urban areas. Surprisingly the overall population of SFU in both communities was 25.2% and it is much less than 67% which is the national average for Nigeria [11]. Despite this low level of SFU exposure, a study in South Western Nigeria demonstrated that there is increased risk of respiratory symptoms and poor lung function test in women using firewood, agric waste and charcoal when compared with those using non biomass fuels [12]. Apart from the harmful effect on human health, the use of solid fuel also has some environmental implications like increase green house gas emission, deforestation and desert encroachment [1, 2]. In order to determine the factors associated with respective energy in urban and rural areas.The six sources of cooking energy were merged into four: solid fuel, kerosene, gas and electricity.

We found the that not owning a dwellings, large household size and low level of education of the respondents were the leading factors associated with the use of solid fuel in the urban household, whereas in the rural household the leading factors were low wealth, education of the respondents and availability of wood and residing in traditional houses.

Education of the respondents was a significant determinant in both urban and rural household. The level of education determines the household income, knowledge attributes and preference of a woman for cleaner fuel. Women who are highly educated are more likely to be engaged in white collar job in which there is limited time for sourcing of solid fuel for cooking. The role of education is consistent with findings in previous studies [13, 14, 20, 25–27].

Ownership of dwelling was not associated with solid fuel use. This result is in contrast to several hypotheses that support the association ownership of dwelling with use solid fuel. Lack of ownership of dwelling may actually be a reflection of the household wealth and poverty in our setting.

The positive association between household size and SFU discovered is in agreement with study in urban areas of Ouagadougou, Burkina Faso and Cameroon [14, 26]. Mekonen et al in Ethiopia reported a different finding that households with more members consumed more electricity and kerosene, but wood and charcoal consumption did not depend on family size [27]. Larger households have more hands to collect firewood from the bush. This free collection of firewood reduces the price of firewood relative to alternative fuel which cannot be obtained freely. It is also assumed to be cheaper to cook for many people using firewood than its alternatives [25–27].

In the rural areas, the inhabitants will readily choose a source of solid fuel like wood and charcoal that are readily available, and less expensive than gas and electricity which are not readily available and are regarded as expensive.

The leading factors associated with the use of kerosene in urban areas was higher level of education of respondent, being married and increased frequency of cooking staple food, while in the rural household it was younger age of the respondent. Higher level of education of the couples in a household often translates to better income and cleaner fuel.

The correlation between residing in modern houses and use of kerosene as fuel is in support of the assumption that a modern type house is an indicator of wealth or the availability of money to support purchases of the more expensive better fuels. The association between marriage, frequency of cooking staple food and utilization of kerosene may be as a result of the wife looking for fast and effective method of regularly cooking for family. Considering the limited amount time needed to meet up with other daily function. In contrast, a study in urban Cameroun found no association between marriage and utilization of kerosene [26].

In the rural areas, the results show that average age has negative statistically significant coefficients with the use of kerosene, thereby agreeing with the theoretical expectation and is comparable with the result in other studies [14, 25, 26].

As regard the use of gas, high household income and wealth were also associated with the use of gas in both the urban and rural areas respectively. The other factors were residing in modern houses, household size and affordability of the fuel in urban areas and kitchen location in the rural areas.

Increase in the household level of wealth is often associated with alleviation of poverty which drives up the household on the energy ladder. The various types of fuels are often conceptualized as forming an “energy ladder” up which households must ascend from solid fuel to non solid fuel as soon as their economic circumstances permit [13, 14, 22].

The use of electricity in the urban areas was associated with higher level of education of respondent, presence of utilities and availability of electricity while in the rural areas it was old age and kitchen location. The association of electricity as a cooking fuel with higher education and presence of utilities may be attributed to increase socioeconomic status which drives the household upward on the energy ladder. We are not surprised at the role of availability of electricity as the supply is inconsistent and erratic in most Nigerian communities. Age was significantly associated with use of electricity, previous studies has revealed age was positively correlated with use of firewood [14, 25, 26].

This study also revealed that household wealth was positively associated with cleaner fuel like gas high in the energy ladder and negatively associated with solid fuel which is lower in energy ladder. Therefore the government and other stakeholders need to promote interventions that will enable low income earner to use higher-quality, lower-emission liquid or gaseous fuels.

Community hygiene education should be directed at influencing choice of household fuel. Hygiene education can play an important role by conveying the value of cleaner kitchens and air to households, may also be as important in reducing the impact of dirty combustion and lack of ventilation as it is in reducing the impact of dirty water and lack of sanitation [17].

The strength of this study is its ability to establish the pattern of household cooking fuel and determine the factors that influences the choice fuel in two different settings which are different in terms of socioeconomic structures. The limitation of the study was the non validation of the survey instrument, small sample size of the study population.

## Conclusion

In conclusion, the prevalence of solid fuel and kerosene use was higher in the rural areas than urban areas while the use gas, electricity and agric waste was higher in the rural areas. The choice of cooking fuel in both rural and urban was influenced by age, education, wealth, income, availability of fuel kitchen location and awareness of the harmful effects of smoke from solid fuel which are all modifiable. The reasons for not using cleaner fuel are more than socioeconomic and technical issues discussed above, other factors influencing the choice of fuel like cultural and perceptual factors should addressed. This study has highlighted the need to reduce poverty to move up the energy ladder, advocate the use of cleaner cooking fuels and improve ventilation particularly in rural areas, towards reducing air pollution and improving lung health.
